# Exploring Microbial Metabolite Receptors in Inflammatory Bowel Disease: An In Silico Analysis of Their Potential Role in Inflammation and Fibrosis

**DOI:** 10.3390/ph17040492

**Published:** 2024-04-12

**Authors:** Michail Spathakis, Nikolas Dovrolis, Eirini Filidou, Leonidas Kandilogiannakis, Gesthimani Tarapatzi, Vassilis Valatas, Ioannis Drygiannakis, Vasilis Paspaliaris, Konstantinos Arvanitidis, Vangelis G. Manolopoulos, George Kolios, Stergios Vradelis

**Affiliations:** 1 Laboratory of Pharmacology, Faculty of Medicine, Democritus University of Thrace, 68100 Alexandroupolis, Greece; mspathak@med.duth.gr (M.S.); efilidou@hotmail.com (E.F.); lkandilo@med.duth.gr (L.K.); mtarapagi@gmail.com (G.T.); valatas@gmail.com (V.V.); karvanit@med.duth.gr (K.A.); emanolop@med.duth.gr (V.G.M.); gkolios@med.duth.gr (G.K.); 2 Individualised Medicine & Pharmacological Research Solutions Center (IMPReS), 68100 Alexandroupolis, Greece; 3 Gastroenterology and Hepatology Research Laboratory, Medical School, University of Crete, 71003 Heraklion, Greece; idrygiannakis@gmail.com; 4 Tithon Biotech Inc., San Diego, CA 92127, USA; bpaspa@tithonbiotech.com; 5 Department of Internal Medicine, University Hospital of Alexandroupolis, Democritus University of Thrace, 68100 Alexandroupolis, Greece; svradeli@med.duth.gr

**Keywords:** microbiota, metabolites, metabolite receptors, inflammatory bowel disease, inflammation

## Abstract

Metabolites produced by dysbiotic intestinal microbiota can influence disease pathophysiology by participating in ligand–receptor interactions. Our aim was to investigate the differential expression of metabolite receptor (MR) genes between inflammatory bowel disease (IBD), healthy individuals (HIs), and disease controls in order to identify possible interactions with inflammatory and fibrotic pathways in the intestine. RNA-sequencing datasets containing 643 Crohn’s disease (CD) patients, 467 ulcerative colitis (UC) patients and 295 HIs, and 4 *Campylobacter jejuni*-infected individuals were retrieved from the Sequence Read Archive, and differential expression was performed using the RaNA-seq online platform. The identified differentially expressed MR genes were used for correlation analysis with up- and downregulated genes in IBD, as well as functional enrichment analysis using a R based pipeline. Overall, 15 MR genes exhibited dysregulated expression in IBD. In inflamed CD, the hydroxycarboxylic acid receptors 2 and 3 (HCAR2, HCAR3) were upregulated and were associated with the recruitment of innate immune cells, while, in the non-inflamed CD ileum, the cannabinoid receptor 1 (CNR1) and the sphingosine-1-phospate receptor 4 (S1PR4) were downregulated and were involved in the regulation of B-cell activation. In inflamed UC, the upregulated receptors HCAR2 and HCAR3 were more closely associated with the process of TH-17 cell differentiation, while the pregnane X receptor (NR1I2) and the transient receptor potential vanilloid 1 (TRPV1) were downregulated and were involved in epithelial barrier maintenance. Our results elucidate the landscape of metabolite receptor expression in IBD, highlighting associations with disease-related functions that could guide the development of new targeted therapies.

## 1. Introduction

Inflammatory bowel disease (IBD) is a chronic inflammatory condition primarily affecting the gastrointestinal tract, with an overall increasing incidence over the last few decades [1]. Crohn’s disease (CD) and ulcerative colitis (UC) are the main clinical manifestations of IBD with a currently undefined pathophysiology [2]. Most recent efforts towards the understanding of these diseases have pointed towards the dysregulation of the intestinal microbiota composition, termed as dysbiosis, being the most plausible cause [3]. Nevertheless, whether dysbiosis is the driving force behind the development of IBD or the result of inflammation-related alterations in the intestinal ecosystem is not yet known [4]. Furthermore, there is no current consensus regarding the exact mechanisms through which the dysbiotic intestinal microbiota influences the pathogenesis of IBD [3].

Even though the gut microbiome is highly variable between individuals [5] and its composition depends on several factors including diet and geographic location [6], the cumulative function of the microbiota at the gene level is remarkably conserved, meaning that it can be considered a “core microbiome” [7]. These conserved functions yield a wide array of metabolites, composing an intestinal microbiota metabolome that far exceeds the human counterpart, and which can serve as a communication medium between the microbial cells and the host [8].

The dysbiosis of the intestinal microbiota in IBD is also reflected in the gut metabolome with the changes in the composition of several metabolite classes, such as sphingolipids and bile acids [9], and these alterations have been observed to have a direct effect on the host’s health. For example, the depletion of short chain fatty acids (SCFAs), which is well-characterized in IBD, deprives colonic epithelial cells of their energy-producing substrate butyrate, and can lead to an impairment of the intestinal epithelial barrier [10], whereas an excess of primary bile acids (PBAs), such as cholic acid, can activate pro-inflammatory pathways in the intestine [11].

To mediate these effects, the host expresses an array of receptors that can bind and respond to different metabolites [12]. However, few studies have explored the expression of these receptors in IBD. We hypothesize that a dysregulation of metabolite receptor gene expression could be driving the development of inflammation and fibrosis in IBD, and, in this study, we offer a comprehensive investigation into metabolite receptor gene expression in IBD. Furthermore, we explore the association between metabolite receptors and other dysregulated genes in IBD, and we provide functional insights through “guilt-by-association” networks. Our results show that metabolite receptor gene expression is indeed perturbed in IBD, and we suggest a strong correlation with immune-modulatory processes, thus aiding the discovery of novel therapeutic target candidates.

## 2. Results

### 2.1. Metabolite Receptor Genes Are Differentially Expressed in Crohn’s Disease and Ulcerative Colitis

**Figure 1:**
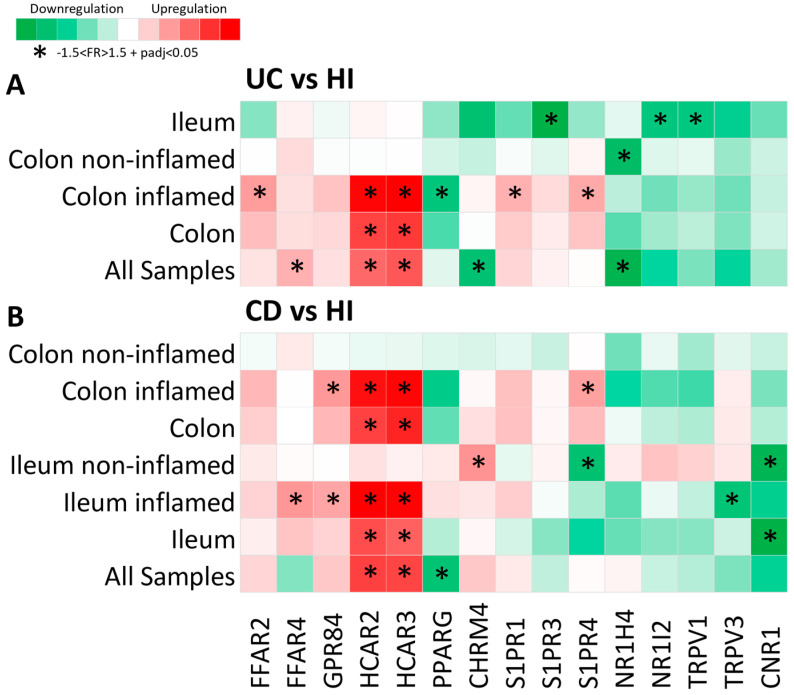
Heat map of metabolite receptor gene expression in (**A**) UC as compared to HI and (**B**) CD as compared to HI. Each row represents a single analysis, including either all samples found in the datasets or subgroups of data from a single location, with further subcategorization regarding inflammation status (inflamed and non-inflamed regions). Each column represents the expression of a single receptor gene in each analysis performed. Criteria for significant up- or downregulation were set as follows: fold regulation between −1.5 and 1.5 and adjusted *p* value < 0.05 (−1.5 < FR > 1.5 + padj < 0.05), and significantly up- or downregulated genes are noted with *.

In order to elucidate the metabolite receptor (MR) expression landscape in IBD, we performed a differential expression analysis of intestinal biopsies from CD and UC patients, comparing their expression to healthy individuals (HI; control group). Of the total of 105 genes that were selected analyzed as microbial metabolite receptor candidates, 15 (14%) exhibited significantly dysregulated expression in IBD, and their differential expression is visualized in Figure 1. A file containing the results of the differential expression of all the genes included in this study can be found in Table S1 in Supplementary Materials.

Overall, of the fifteen MR genes that exhibited dysregulated expression in IBD, six were upregulated in UC, six were downregulated in UC, five were upregulated in CD, and three were downregulated in CD. Interestingly, one MR gene, the sphingosine 1-phosphate receptor 4 (S1PR4), was found to be downregulated in the non-inflamed CD ileum and upregulated in the inflamed CD colon when compared to the corresponding HI samples (Figure 1A,B).

More specifically, the hydroxycarboxylic acid receptors 2 and 3 (HCAR2 and HCAR3) were significantly upregulated in both UC (HCAR2: FR = 2.42, padj = 4.72 × 10^29^; HCAR3: FR = 2.65, padj = 3.67 × 10^37^) and CD (HCAR2: FR = 2.41, padj = 1.79 × 10^60^; HCAR3: FR = 2.36, padj = 1.73 × 10^73^), and their upregulation was highly correlated with inflammation, as in the samples from non-inflamed regions, where there was no observable up- or downregulation of these genes (Figure 1A,B). The free fatty acid receptor 2 (FFAR2) was upregulated in the inflamed UC colon (FR = 1.79, padj = 1.79 × 10^46^) (Figure 1A), and the free fatty acid receptor 4 (FFAR4) exhibited upregulated expression in UC (FR = 1.59, padj = 1.8 × 10^13^) and in the inflamed CD ileum (FR = 1.63, padj = 0.001) (Figure 1A,B). The G-protein-coupled receptor 84 (GPR84) was also upregulated in the inflamed CD ileum and colon (FR = 1.52, padj = 2.76 × 10^9^) (Figure 1B), whereas the sphingosine 1-phosphate receptor 1 (S1PR1) was upregulated in the inflamed UC colon (FR = 1.58, padj = 3.93 × 10^30^) (Figure 1A). Furthermore, the cholinergic receptor muscarinic 4 (CHRM4) was upregulated in samples from the non-inflamed CD ileum (FR = 1.66, padj = 9.62 × 10^7^) and was downregulated in UC (FR = −1.64, padj = 2.83 × 10^13^) (Figure 1A,B). The S1PR4 was also upregulated in the inflamed UC colonic mucosa (FR = 1.67, padj = 1.79 × 10^22^) (Figure 1A). The peroxisome proliferator activated receptor γ (PPARG) was found to be downregulated in CD (FR = −1.56, padj = 5.32 × 10^11^), with a greater tendency of downregulation in the inflamed colon, as well as in the inflamed UC colon (FR = −1.57, padj = 1.12 × 10^13^) (Figure 1A,B). The nuclear receptor 1H4 (NR1H4), also known as the farnesoid X receptor (FXR), was significantly downregulated in UC, with greater specificity in the non-inflamed colonic mucosa (FR = −1.70, padj = 0.032) (Figure 1A), whereas the sphingosine 1-phosphate receptor 3 (S1PR3), the nuclear receptor 1I2 (NR1I2), and the transient receptor potential vanilloid 1 (TRPV1) were downregulated in the UC ileum when compared to the HI ileum (S1PR3: FR = −1.87, padj = 0.0001; NR1I2: FR = −1.57, padj = 0.042; TRPV1: FR = −1.55, padj = 0.001) (Figure 1A). Finally, the transient receptor potential vanilloid 3 (TRPV3) was downregulated in the inflamed CD ileum (FR = −1.55, padj = 1.72 × 10^13^), and the cannabinoid receptor 1 (CNR1) was downregulated in the CD ileum; more specifically, it exhibited significant downregulation in the non-inflamed ileal regions as compared to HI ileum (FR = −1.72, padj = 6.77 × 10^7^) (Figure 1B).

### 2.2. Expression of Metabolite Receptor Genes Is Associated with Inflammation in Inflammatory Bowel Disease

**Figure 2:**
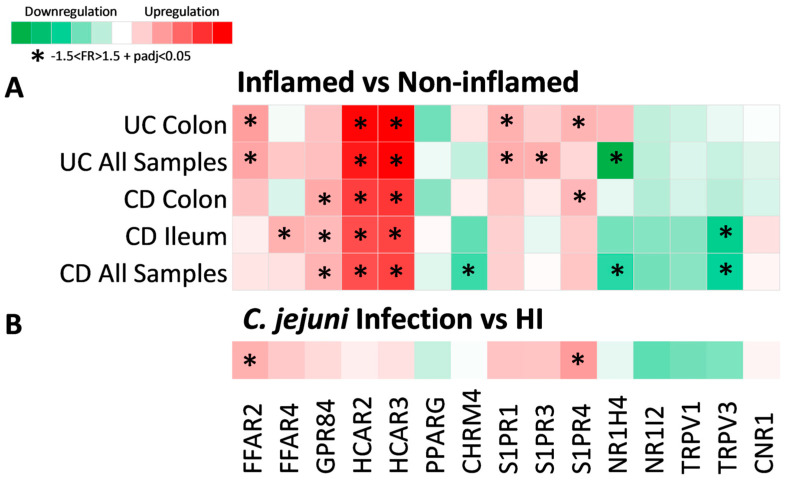
Heat map of metabolite receptor gene expression in (**A**) inflamed compared to non-inflamed intestinal regions and (**B**) *C. jejuni* infection compared to HI. Each row represents a single analysis, including subgroups of data from a single location. Each column represents the expression of a single receptor gene in each analysis performed. Criteria for significant up- or downregulation were set as follows: fold regulation between −1.5 and 1.5 and adjusted *p* value < 0.05 (−1.5 < FR > 1.5 + padj < 0.05), and significantly up- or downregulated genes are noted with *.

The results of our initial analysis indicated that the differential expression of MRs between IBD patients and HIs is influenced by disease activity, thus we proceeded to investigate whether MR genes are differentially expressed between inflamed and non-inflamed regions of the intestine within CD and UC. As 15 of the 104 included MR genes exhibited the significant dysregulation of mRNA expression in IBD, we focused on these genes for our downstream analysis; their differential expression between inflamed and non-inflamed regions in UC and CD is shown in Figure 2A.

HCAR2 and HCAR3 were significantly upregulated in the inflamed intestinal regions of both CD (HCR2: FR = 2.79, padj = 5.45 × 10^20^; HCAR3: FR = 3.00, padj = 2.6 × 10^35^) and UC (HCAR2: FR = 3.73, padj = 5.31 × 10^15^; HCAR3: FR = 4.28, padj = 3.6 × 10^25^), independent of location, indicating that the mRNA expression of these receptors is highly influenced by inflammation in IBD. The receptor GPR84 was also significantly upregulated in inflamed CD (FR = 1.58, padj = 3.26 × 10^30^) in both the ileum and the colon. Furthermore, FFAR2 and S1PR1 were significantly increased in the inflamed UC colon (FFAR2: FR = 1.78, padj = 1.1 × 10^29^; S1PR1: FR = 1.60, padj = 8.1 × 10^21^), whereas FFAR4 was significantly upregulated only in the CD inflamed ileum (FR = 1.60, padj = 2.36 × 10^18^). On the other hand, S1PR3 was upregulated In inflamed UC (FR = 1.61, padj = 2.44 × 10^16^), without meeting our criteria of significance when only the colonic samples were tested. The same applied to CHRM4 and NR1H4, the downregulation of which was significant in inflamed CD (CHRM4: FR = −1.58, padj = 7.09 × 10^5^) and both inflamed CD (NR1H4: FR = −1.62, padj = 0.001) and UC (NR1H4: FR = −2.58, padj = 1.05 × 10^12^), respectively, only when samples from both the colon and ileum were analyzed together, which could be due to differences in the expression of those receptors between the ileal and colonic tissue. Finally, the expression of TRPV3 was significantly decreased in the inflamed CD ileum (FR = −1.82, padj = 2.34 × 10^14^), whereas no significant differences in the expression of NR1I2, TRPV1, or CNR1 were detected between inflamed and non-inflamed samples.

Additionally, in order to further explore the effect of inflammation independently of IBD, we performed a differential expression analysis using a dataset derived from *Campylobacter jejuni*-infected individuals (Figure 2B). Of the aforementioned receptors that were upregulated in the inflamed mucosa of IBD, only FFAR2 (FR = 1.60, padj = 0.029) and S1PR4 (FR = 1.81, padj = 0.025) were found to be upregulated in colonic samples of *C. jejuni*-infected individuals when compared to hIs. The rest of the receptors exhibited a tendency towards upregulation or downregulation that did not reach significance, which could indicate that the upregulation of these genes is more specific to the inflammation of IBD.

### 2.3. Expression of Metabolite Receptors Differs between Colon and Terminal Ileum

**Figure 3:**
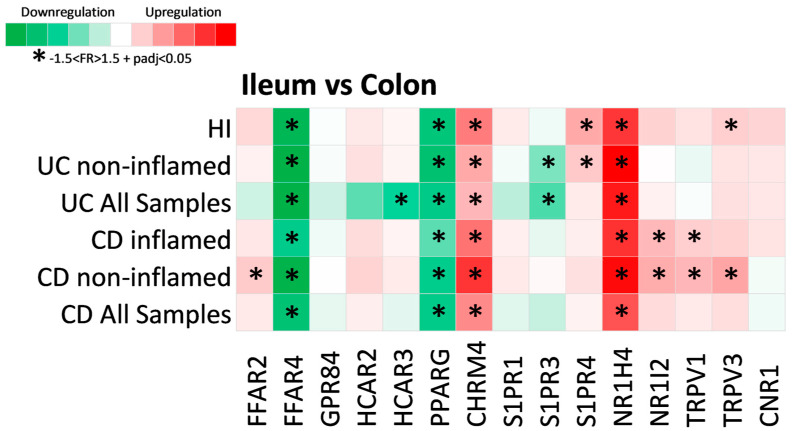
Heat map of metabolite receptor gene expression in the ileum compared to the colon. Each row represents a single analysis, including either all samples found in the datasets or subgroups of data from subcategorization regarding inflammation status (inflamed and non-inflamed regions). Each column represents the expression of a single receptor gene in each analysis performed. Criteria for significant up- or downregulation were set as fold regulation between −1.5 and 1.5 and adjusted *p* value < 0.05 (−1.5 < FR > 1.5 + padj < 0.05), and significantly up- or downregulated genes are noted with *.

Subsequently, we explored whether MR genes are differentially expressed between the ileal and colonic tissue of IBD patients and hIs, and the results of the analysis are shown in Figure 3.

Of the fifteen differentially expressed MR genes in IBD, four were found to be significantly associated with intestinal anatomic location. More specifically, the expression of FFAR4 (CD: FR = −4.01, padj = 1.52 × 10^82^; UC: FR = −5.94, padj = 1.1 × 10^49^; HI: FR = −5.30, padj = 2.53 × 10^74^) and PPARG (CD: FR = −3.31, padj = 6.3 × 10^113^; UC: FR = −3.36, padj = 1.1 × 10^53^; HI: FR = 3.73, padj = 1.7 × 10^122^) was downregulated in the ileum when compared to colon, whereas CHRM4 (CD: FR = 2.75, padj = 6.11 × 10^61^; UC: FR = 1.88, padj = 8.53 × 10^21^; HI: FR = 3.09, padj = 2.6 × 10^37^) and NR1H4 (CD: FR = 4.38, padj = 1.06 × 10^86^; UC: FR = 6.75, padj = 6.48 × 10^39^; HI: FR = 5.39, padj = 9.27 × 10^36^) were significantly upregulated in the UC, CD, and HI ileum. Interestingly, S1PR3 was significantly downregulated in the ileum compared to the UC colon, even when only non-inflamed samples were tested (FR = −1.92, padj = 7.28 × 10^6^), but this was not observed in the ileum of hIs. The same was observed for the expression of NR1I2 and TRPV1, which were found to be upregulated in the CD ileum when compared to the colon in both inflamed (NR1I2: FR = 1.85, padj = 4.58 × 10^20^; TRPV1: FR = 1.53, padj = 1.6 × 10^33^) and non-inflamed samples (NR1I2: FR = 2.06, padj = 6.03 × 10^50^; TRPV1: FR = 1.88, padj = 4.87 × 10^48^), but not in HIs, leading us to assume that these differences may either reflect a specific effect of IBD, or be associated with their pathogenesis. Similarly, the expression of S1PR4 was upregulated in the ileum of HIs when compared to the colon (FR = 2.11, padj = 1.02 × 10^46^), as well as in the ileum of the non-inflamed UC (FR = 1.61, padj = 1.82 × 10^31^); however, this was not observed for the CD ileum, whereas TRPV3 was upregulated in the ileum of HIs (FR = 1.53, padj = 5.39 × 10^19^) and the non-inflamed CD ileum (FR = 2.18, padj = 3.15 × 10^20^). Finally, FFAR2 was upregulated in the ileum of non-inflamed CD (FR = 1.59, padj = 1.51 × 10^78^), but not in HIs, and none of GPR84, HCAR2, HCAR3, S1PR1, or CNR1 were found to be dependent on their location. HCAR3 was significantly downregulated in the UC ileum (FR = −2.77, padj = 0.029), which could be an effect of inflammation, as this trend was not observed when only the non-inflamed samples were analyzed, and also HCAR3 was significantly upregulated in the inflamed UC colon (Figure 2).

### 2.4. Metabolite Receptor Gene Expression Exhibits Differential Correlation with Up- and Downregulated Genes in Inflammatory Bowel Disease and Healthy Individuals

**Figure 4:**
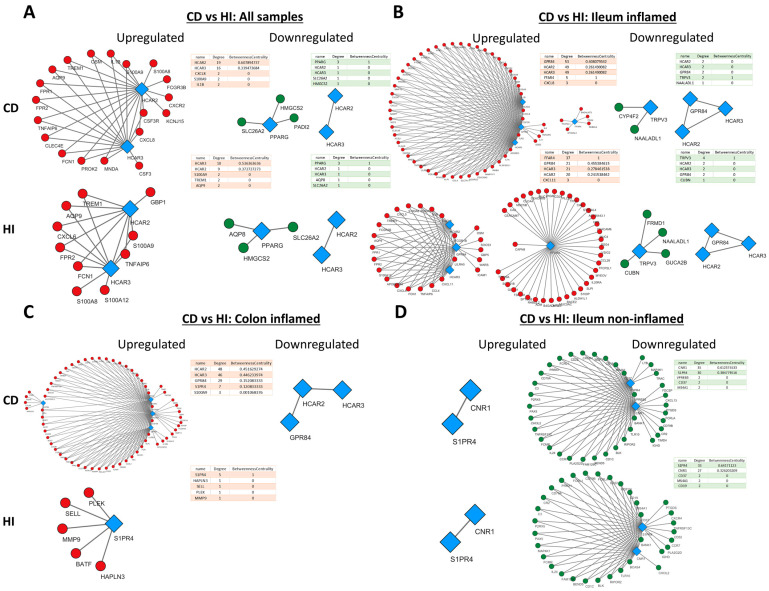
Expression correlation networks of MRs with up- and downregulated genes in CD. Each tetrad represents data from a single analysis and consists of four networks. In each tetrad, MR connections with up- (first column) and downregulated (second column) genes in patients with CD are shown on the upper row, and HIs are shown on the lower row. Specifically, (**A**) Crohn’s Disease versus Healthy Individuals regardless of inflammation status or disease location (**B**) Crohn’s Disease versus Healthy Individuals in the inflamed terminal ileum (**C**) Crohn’s Disease versus Healthy Individuals in the inflamed colon (**D**) Crohn’s Disease versus Healthy Individuals in the non-inflamed terminal ileum. Red circular nodes represent upregulated genes, green circular nodes represent downregulated genes, and blue rhomboidal nodes represent MR genes. Only connections with strongly correlated genes are shown (r > 0.8).

**Figure 5:**
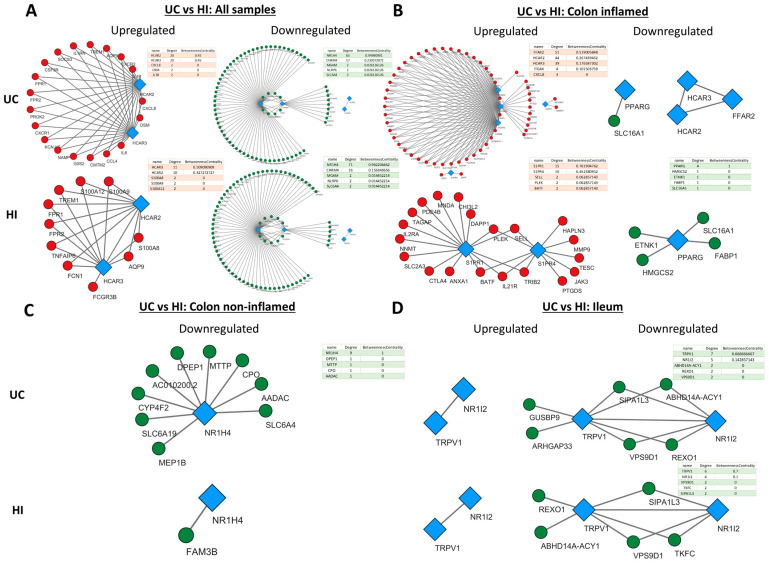
Expression correlation networks of MRs with up- and downregulated genes in UC. Each tetrad represents data from a single analysis and consists of four networks. In each tetrad, MR connections with up- (first column) and downregulated (second column) genes in patients with UC are shown on the upper row, and HIs are shown on the lower row. Specifically, (**A**) Ulcerative Colitis versus Healthy Individuals regardless of inflammation status or disease location (**B**) Ulcerative Colitis versus Healthy Individuals in the inflamed colon (**C**) Ulcerative Colitis versus Healthy Individuals in the non-inflamed colon (**D**) Ulcerative Colitis versus Healthy Individuals in the non-inflamed terminal ileum. Red circular nodes represent upregulated genes, green circular nodes represent downregulated genes, and blue rhomboidal nodes represent MR genes. Only connections with strongly correlated genes are shown (r > 0.8).

Having established the differential expressions of MRs between IBD and HIs, we subsequently looked into the associations of MR expression with genes that exhibit dysregulated expression in CD and UC by performing an expression correlation analysis using Spearman’s method of the correlation coefficient. For each of our analyses, the MRs with dysregulated expression in IBD were chosen, and their strongly correlated (r > 0.8) up- or downregulated genes in CD and UC are shown in Figure 4 and Figure 5, respectively. Networks were created separately for CD/UC and HI in order to uncover state-specific associations between MRs and genes with dysregulated expression in IBD.

In general, the networks of upregulated genes had the most nodes and edges in CD in comparison to HI, whereas the number and pattern of nodes and edges amongst downregulated genes remained more or less the same in CD and HIs. In addition, HCAR2, HCAR3, and GPR84 exhibited strong correlation with each other and a large number of upregulated genes (Figure 4A–C), and the same was observed between S1PR4, CNR1, and downregulated genes (Figure 4D).

More specifically, when all samples were included in the analysis (Figure 4A), HCAR2 was associated with the most upregulated genes in CD (degree = 19), while HCAR3 had the most connections with upregulated genes in His (degree = 10). In the inflamed CD ileum (Figure 4B), GPR84 exhibited the highest connectivity with upregulated genes (degree = 53), followed closely by HCAR2 and HCAR3, with all of their connections being shared (degree = 49), and FFAR4 (degree = 5). Interestingly, in the ileum of HIs, FFAR4 assumed the role of the hub MR (degree = 37), while GPR84, HCAR3, and HCAR2 formed a much smaller network with upregulated genes (degree = 21, 21, and 20, respectively). Moreover, there were no common connections between FFAR4 and the rest of GPR84, HCAR2, and HCAR3, which formed an independent, close-knight network, proposing that these genes may participate in different functions. In the inflamed CD colon (Figure 4C), the four upregulated receptors GPR84, HCAR2, HCAR3, and S1PR4 formed a single network with upregulated genes, again with HCAR2 exhibiting the highest connectivity (degree = 48), followed by HCAR3 ((degree = 46), GPR84 (degree = 29), and S1PR4 (degree = 7). In contrast, in HIs, the receptors GPR84, HCAR2, and HCAR3 did not exhibit any strong correlations with CD-upregulated genes, and only S1PR4 formed a network, although with different genes (degree = 5). Regarding the downregulated genes, in the analysis including all samples, only PARG showed an association with downregulated genes in both CD and HIs (degree = 3) (Figure 4A), whereas, in the inflamed CD ileum, TRPV3 participated in networks with downregulated genes in CD (degree = 2) and HIs (degree = 4), while the expression of HCAR2, HCAR3, and GPR84 only correlated with each other’s (Figure 4B). In the inflamed CD colon, there were no strongly correlated downregulated genes with MRs. Finally, in the non-inflamed CD ileum (Figure 4D), the downregulated MRs S1PR4 and CNR1 strongly correlated with other downregulated genes, with CNR1 having the most connections in CD (degree = 35), and S1PR4 emerging as the most interconnected MR in HIs (degree = 33).

HCAR2 and HCAR3 exhibited the same pattern of connections with upregulated genes in UC. When all samples were pooled together (Figure 5A), HCAR2 and HCAR3 had the same connections in CD (degree = 20), whereas HCAR3 had a slightly greater connectivity than HCAR2 in HIs (degree = 11 over 10). In the inflamed UC colon (Figure 5B), both HCRA2 and HCAR3 strongly correlated with a large number of upregulated genes; however, FFAR2 showed the most connections (degree = 51). Interestingly, in the colon of HIs, there were no strong connections between these receptors and upregulated genes, and S1PR1 and S1PR4 emerged as the hub MRs in this group (degree = 15 and 10, respectively). On the other hand, the receptors NR1H4 and CHRM4 were strongly correlated with several downregulated genes in UC, with NR1H4 having the most connections in both UC (degree = 65) and HIs (degree = 71) (Figure 5A). In the inflamed UC colon (Figure 5B), the expression of PPARG correlated with downregulated genes and formed a small network in His (degree = 4). In the non-inflamed UC colon (Figure 5C), NR1H4 strongly correlated with downregulated UC genes, although, at a lower degree (degree = 9) when compared to the pooled-sample analysis. Lastly, in the UC ileum (Figure 5D), TRPV1 and NR1I2 strongly correlated with a small number of downregulated genes in both UC and HIs, with TRPV1 showed the most connections (degree = 7 and 6, respectively).

### 2.5. Metabolite Receptors Associate with the Processes of Inflammation and Fibrosis in Crohn’s Disease and Ulcerative Colitis

To further elucidate the potential involvement of each MR in the pathogenetic processes of IBD, we continued to perform a functional enrichment analysis of the strongly correlated up- and downregulated genes with each MR gene. This process yielded a set of biological processes which were strongly correlated with the expression of each MR in the specific analysis. The tables featuring the GO terms of these biological processes, arranged in a descending order based on their combined score, are shown in Supplementary Figure S1 for the analyses comparing CD with His, and in Supplementary Figure S2 for the analyses comparing UC with His, in Supplementary Materials.

As expected from their expression in inflamed mucosa, the upregulated genes in CD that were strongly correlated with HCAR2 and HCAR3 were also mostly involved in the development of the inflammatory response and, more specifically, with leucocyte aggregation (GO:0070486), the chemotaxis and migration of neutrophils (GO:0030593 and GO:1990266), and the activation of granulocytes (GO:0036230). The enrichment in these functions was also observed in the HI group, even though in the analysis including all samples, HCAR2 was associated with more homeostatic functions in His, such as autocrine signaling (GO:0035425) and astrocyte differentiation (GO:0048708) (Supplementary Figure S1A,B). The same pattern was observed for GPR84, the associated genes of which were also involved in the chemotaxis of granulocytes (GO:0071621) and the development of an immune response in CD in both the inflamed ileum and the inflamed colon (Supplementary Figure S1C). Interestingly, in the ileum of His, the negative regulation of hormone secretion (GO:0046888) gained importance, as did the regulation of interleukin-1-mediated signaling pathways (GO:2000659) in the inflamed colon of CD patients (Supplementary Figure S1C). In the inflamed CD ileum, the function of FFAR4-associated upregulated genes was also enriched for immune response-related processes, such as the positive regulation of leucocyte tethering or rolling (GO:1903238), as well as for the processes of hemostasis (GO:1900048) and coagulation (GO:0050820). In the ileum of His, on the other hand, FFAR4 exhibited enriched involvement in homeostatic processes, including the regulation of monoatomic anion transport (GO:0044070), the positive regulation of the autophagy of mitochondrion in response to mitochondrial depolarization (GO:1904925), and the negative regulation of Anoikis (GO:2000811) (Supplementary Figure S1D). In the inflamed CD colon, S1PR4 was associated with the extracellular matrix regulation processes, the positive regulation of extracellular matrix disassembly (GO:009091), and actin-mediated cell contraction (GO:0070252), as well as with the processes of hematopoietic stem cell differentiation (GO:0060218) and folic acid transport (GO:0015884), whereas in HIs, cell signaling-related processes showed a higher combined score (Supplementary Figure S1E). In contrast, in the non-inflamed ileum of CD patients and HIs, S1PR4 and CNR1 were associated with downregulated genes which were mainly involved in B-cell proliferation (GO:0042100), activation (GO:0050864), and signaling (GO:0050853) (Supplementary Figure S1F,G). In the pooled-sample analysis of CD patients, PPARG was strongly correlated with downregulated genes which controlled ketone body biosynthesis and metabolism (GO:0046951 and GO:1902224), as well as the negative regulation of lymphocyte migration and chemotaxis (GO:2000402), whereas, in the corresponding HI samples, PPARG was associated with the same homeostatic processes, while there was no association with genes involved in immune-related processes (Supplementary Figure S1H). Finally, in the ileum of HIs, the downregulated genes of the inflamed CD ileum that were correlated with TRPV3 participated in the homeostatic processes of cobalamin metabolism and transport (GO:0009235 and GO:0015889), tetrapyrrole metabolism (GO:0033013), and the positive regulation of Hippo signaling (GO:0035332) (Supplementary Figure S1I).

In UC, the same pattern was observed for HCAR2 and HCAR3, which were associated with upregulated genes which were mainly involved in the regulation of an inflammatory response through the regulation of interleukin-1-mediated signaling (GO:2000659) and TH-17 differentiation (GO:0072539) (Supplementary Figure S2A,B). In the inflamed UC colon, FFAR2 was associated with the same inflammatory response-related processes as HCAR2 and HCAR3, but also with the positive regulation of unsaturated fatty acid biosynthetic processes (GO:2001280) (Supplementary Figure S2C). In the colon of His, but not in UC, the sphingosine-1-phospahate receptors S1PR1 were correlated with the UC-upregulated genes involved in the restriction of the inflammatory response, as well as in homeostatic processes (Supplementary Figure S2D). More specifically, S1PR1 was associated with the negative regulation of B-cell proliferation and activation (GO:0030889 and GO:0050869), as well as the regulation of muscle relaxation (GO:1901078) and the metabolism of L-ascorbic acid (GO:0019852) (Supplementary Figure S2D). On the other hand, in both inflamed UC colons and the colons of HIs, S1PR4 was associated with pro-inflammatory processes, such as the negative regulation of interleukin-10 production (GO:0032693), the intetrleukin-4-mediated signaling pathway (GO:0035771), and the regulation of T-helper 1 cell differentiation (GO:0045625) (Supplementary Figure S2E). In the pooled-sample analysis, CHRM4 and NR1H4 strongly correlated with several downregulated genes in UC, which were mostly common between UC and HIs (Supplementary Figure S2A). The functional enrichment for the CHRM4 correlated genes revealed its involvement in metabolic processes such as proline metabolism (GO:0006560) and the urea cycle (GO:0000050), the transport of nucleobases (GO:0015851), glycolipids (GO:0046836), and phospholipids (GO:2001138), as well as the regulation of intestinal nervous system processes such as the negative regulation of excitatory postsynaptic potential (GO:0090394) and neurotransmitter reuptake (GO:0098810) (Supplementary Figure S2F). NR1H4 was also associated with metabolic processes, mainly the urea cycle (GO:0000050) and fructose transport and metabolism (GO:0015755 and GO:0006000), as well as the regulation of systemic arterial pressure through the renin-angiotensin system (GO:0003081) (Supplementary Figure S2G). In the ileum of both UC patients and HIs, the receptors NR1I2 and TRPV1 significantly correlated with the UC-downregulated genes associated with the regulation of intestinal epithelial cell morphogenesis (GO:0003382), development (GO:0002064), and polarization (GO:0090162), even though, in HIs, there was also a correlation with the processes involved in responses against pathogens, such as the regulation of the MDA-5 signaling pathway (GO:0039533) and the negative regulation of the viral-induced cytoplasmic pattern recognition receptor signaling pathway (GO:0039532) (Supplementary Figure S2H,I). Similarly to CD, PPARG strongly correlated with UC-downregulated genes in the colons of His, which were involved in metabolic processes such as succinate transport (GO:0015744), ketone body biosynthesis and metabolism (GO:0046951 and GO:1902224), and the biosynthesis of phosphatidylethanolamine (GO:0006646) (Supplementary Figure S2J). Finally, in the non-inflamed UC colon, NR1H4 was again associated with the regulation of systemic arterial pressure (GO:0001977), as well as the metabolism of lipoproteins (GO:0042157), heterocyclic compounds (GO:0046700), long-chain fatty acids (GO:0042758), and processes involved in responses to nitrosative stress (GO:1902170 and GO:0071732) (Supplementary Figure S2K).

## 3. Discussion

In this study, we investigated the expression of metabolite receptor genes in the intestine of patients with Crohn’s disease and ulcerative colitis as compared to healthy individuals, and we identified 17 of these genes to be dysregulated in disease, depending on inflammation and intestinal location. Furthermore, we explored the association of these genes with other up- and downregulated genes in CD and UC, as well as the disease-dysregulated biological processes, in which they might be involved.

Of the 17 dysregulated metabolite receptors, HCAR2 and HCAR3 exhibited the most significant and consistent upregulation in IBD located in the inflamed areas of the intestine. This is in line with the findings of Nuzzo et al., who reported an upregulated expression of HCAR2 in biopsies from IBD patients [13], as well as with Wnorowski et al. and Kaddoura et al., who both found HCAR3 to be upregulated in IBD through the use of microarray data [14,15].

The strong dependence of the IBD-related overexpression of HCAR2 and HCAR3 on the presence of inflammation observed in our findings indicates either that the transcription of these genes is influenced by inflammation, or that they are expressed in the immune cells of the inflamed tissue. Indeed, both these receptors are expressed by various innate immune cells, including macrophages, neutrophils, and dendritic cells, as well as the colonic epithelium, and their expression in immune cells was also found to be inducible by cytokines [16]. Interestingly, this finding was not repeated when the expression of the inflamed colonic mucosa of *C. jejuni*-infected patients was compared to that of healthy individuals, where HCAR2 and HCAR3 followed the same tendency of overexpression, though without reaching significance, thus indicating a possible role of these receptors specific to the development of inflammation in IBD.

The naturally occurring ligands for HCAR2 are the SCFA butyrate and its hydroxylated derivative, β-hydroxybutyrate, as well as nicotinic acid and the carboxylic acids pentanoate, hexanoate, heptanoate, and octanoate [17], whereas HCAR3 has been found to be activated through the binding of the hydroxycarboxylic acid 3-hydroxyoctanoic acid and the tryptophan metabolite kynurenic acid [18]. The activation of HCAR2 by butyrate has been demonstrated to protect against experimental colitis through suppressing the inflammatory response in immune cells and reinforcing the intestinal epithelial barrier [19]. Additionally, HCAR3 overexpression has been found to be decreased in response to a successful anti-TNF-α treatment [14,15], and its activation in innate immune cells has been reported to stimulate their migration [20], and downregulate the expression of proinflammatory cytokines [21], thus suggesting a possible protective/anti-inflammatory role of this receptor. However, the exact role of HACR3 in IBD pathophysiology is still unexplored.

Moreover, we performed an expression correlation analysis and a functional enrichment analysis of the highly correlated IBD-upregulated genes for each receptor. Our results show a strong correlation of HCAR2 and HCAR3 with several upregulated genes in CD and UC, and an association with the processes involved in the development of an acute inflammatory response, as well as the attraction and activation of innate immune cells. This finding, combined with the strong correlation between the expression of HCAR2 and HCAR3, supports the notion of the innate immune cell localization of these receptors that may explain the strong connection between their overexpression and active inflammation in IBD. In addition, we report associations with the known functions of HCAR2 and HCAR3, such as neutrophil migration [20], the autocrine signaling and negative regulation of hormone secretion [22,23], and the positive regulation of IL-18 production [19], together with a novel association of both receptors with the processes of TH-17 cell lineage commitment and differentiation in CD and UC.

Furthermore, the free fatty acid-sensing receptors FFAR2, FFAR4, and GPR84 were also found to be upregulated in IBD, with FFAR2 exhibiting upregulated expression in the inflamed UC colon, FFAR4 in the inflamed CD ileum and in UC, and GPR84 in the inflamed CD ileum and colon. FFAR2 has been reported to be upregulated in active IBD, a change that may reflect the recruitment of innate immune cells and its upregulated expression via TNF-α [24]. This is in agreement with our results that show the significant upregulation of FFAR2 expression during both IBD- and non-IBD-related inflammation (Figure 2). The same applies to the expression of FFAR4, which is reported to be upregulated in CD as a direct effect of TNF-α [25], whereas, as far as we are concerned, no data exist regarding its expression in UC. Interestingly, we found that FFAR4 is upregulated in the colon of both IBD patients and healthy individuals, and that the inflammation-related overexpression of this receptor in CD is restricted to the ileum; this could indicate that an abnormal upregulation of this gene in the ileum may be important for disease development, though experimental data are needed to support this hypothesis. Finally, the upregulation of GPR84 in patients with active UC has been reported, and this correlated with endoscopic disease activity [26]. However, there are no previous reports in the literature showing an increased expression of this gene in inflamed CD, as our results indicated.

Similarly to HCAR2, FFAR2 is a receptor for microbiota-produced SCFAs, with acetate and propionate being the most potent ligands [17]. The activation of FFAR2 by SCFAs has been demonstrated to exert anti-inflammatory properties and to confer protection against experimental colitis [27]. The results of our functional enrichment analysis suggest a possible role of this receptor in the regulation of leucocyte trafficking on the intestine, as well as agreeing with previous reports showing that FFAR2^+^ polymorphonuclear infiltration in active CD is dependent on dietary fiber consumption [28]. Furthermore, our results also suggest an involvement of this receptor in the inflammatory processes of prostaglandin biosynthesis and IL-1-mediated signaling, as well as the TH-17 cell differentiation, which may reflect the variety of the physiological roles of FFAR2 in different cell types that remain to be explored.

FFAR4 is a receptor for ω-3 long chain fatty acids, and has been experimentally shown to be activated by α-linolenic acid and other ω-3 fatty acids, such as docosahexaenoic acid and eicosapentaenoic acid [29]. Typically, ω-3 fatty acids are ingested via a normal diet [30]; however, the intestinal microbiota also bear the ability to metabolize fatty acids into new bioactive forms that can activate FFAR4 [31]. The activation of FFRA4 by ω-3 fatty acids has been observed to protect against colitis development though actions on both the intestinal epithelium [32] and the intestinal immune cells [33]. Here, we show that FFAR4 in CD is correlated with different genes in the ileum of CD patients and healthy individuals, leading to the association of this receptor with separate functions in the two groups. Interestingly, we also found an association of this receptor with the processes of mitophagy (previously reported in the literature by Chen J. et al. [34]) and anoikis in healthy individuals, as well as with the promotion of hemostasis and coagulation in CD, findings that warrant further investigation.

Finally, GPR84 is activated by medium chain fatty acids and their hydroxylated derivatives [35], and, although medium chain fatty acid concentration in the intestine is heavily dependent on diet, their concentration can also be modulated by the composition of the intestinal microbiota [36]. GPR84 is expressed in immune cells, and this is generally considered to exert pro-inflammatory actions in the intestine [26]. This is in agreement with the results of our functional enrichment analysis, where GPR84 was associated with CD-upregulated genes involved in the development of an acute inflammatory response, similar to HCAR2 and HCAR3.

Regarding the metabolite receptors exhibiting downregulated expression in IBD, we found PPAR-γ to be significantly downregulated in both CD and UC, with a strong dependence on inflammation, which could be attributed to the known downregulation of this receptor via pro-inflammatory cytokines [37]. Several studies so far have linked the impaired expression of PPAR-γ with the development and the clinical course of UC [38,39]; however, relatively little data exist on its expression in CD and its possible impact of its downregulation in this state [40]. A wide range of long chain fatty acids and conjugates have been proposed as ligands for PPAR-γ [41], and its activation has been shown to ameliorate inflammation and protect against colitis in IBD [42]. Interestingly, the expression of PPAR-γ in the colon is enhanced by the intestinal microbiota [38], and, in reverse, the activation of this receptor can influence its composition [42], thus creating a cycle of host–microbiota interactions with relevant implications on IBD pathophysiology.

Moreover, the bile acid-sensing receptors NR1H4 (FXR) and NR1I2 (PXR) were downregulated in both the UC colon and ileum, respectively, and both receptors have previously been reported to be downregulated in the intestinal epithelium of active CD and UC [43]. A dysregulation of bile acid metabolism has been described in IBD patients, with a reduction in secondary bile acids and an increase in primary bile acid contents in stools [9]. FXR is activated by both primary and secondary bile acids, with the most potent agonist being the primary bile acid chenodeoxycholic acid (CDCA) [44]. The upregulated expression of FXR in the ileum, when compared to the colon in IBD patients and HIs, is consistent with the findings of Inagaki et al., who reported the greater mRNA expression of this receptor in the ileum of mice when compared to other intestinal sites [45]. Our results also indicate that FXR expression is downregulated by inflammation, and indeed that TNF-α and IL-1α treatments have been reported to repress the expression of FXR in Hep3B cells in vitro [46]; however, the fact that the observed FXR downregulation in UC was greater when comparing the non-inflamed colonic mucosa of UC patients with HIs suggests that the dysregulation of this gene may not just be an effect of inflammatory stimulation.

Interestingly, PXR, together with S1PR3 and TRPV1, exhibited a downregulated expression, localized in the ileum of UC patients. Considering the fact that Shakhnovich et al. have reported a downregulated expression of PXR in sites of active inflammation in CD, potentially indicating a modulation of PXR expression by inflammation [47], we could assume that the downregulation of these receptors in the UC ileum could be the result of inflammation, developing due to the regurgitation of colonic content into the ileum (backwash ileitis) [48]. However, experimental data are needed to support this hypothesis. PXR is a receptor for bile acids, as well as for several xenobiotic compounds [49], with a recently documented anti-inflammatory and anti-fibrotic effect in DSS colitis [50]. Here, we discovered that PXR expression highly corelated with the downregulated genes involved in epithelial cell homeostasis in UC, an integral part of the intestinal immune barrier, the breakdown of which has been implicated in the pathophysiology of IBD [51]. This is also supported by the findings of Terc et al., who reported that PXR activation promotes the migration of epithelial cells and intestinal wound healing after colitis [51].

The endocannabinoid system and its interactions with the intestinal microbiota are increasingly drawing attention as a potential therapeutic target in IBD [52]. The classical cannabinoid receptor CNR1, as well as the transient receptor potential channels TRPV1 and TRPV3 are considered targets for endocannabinoid compounds [53], and, in this study, we show a downregulation of these receptors in IBD. TRPV1 is the best-studied receptor of the TRP subfamily in IBD. Studies regarding its expression in IBD have yielded various results, with some reporting increased expression in IBD [54,55], while other demonstrated the downregulation of this receptor in IBD [56,57], which may reflect variations in the methodology, inflammation status, and cell type investigated. Interestingly, most studies have explored the expression of TRPV1 in colonic tissue, while data regarding its expression in the ileum are sparse. McVey et al. have demonstrated that the administration of the endocannabinoid anandamide in the ileum of rats induces ileitis in a TRPV1-dependent manner [58], and a gain of the function mutation of this receptor also enhances susceptibility to experimental colitis [59]. However, Zhang et al. have recently showed that TRPV1 activation protects against inflammation and regulates intestinal homeostasis via an interplay with the microbiota [56]. Thus, further studies are needed to establish the role of TRPV1 in intestinal inflammation and IBD.

In this study, we also identified that TRPV3 and CNR1 were downregulated in the inflamed and non-inflamed ileum of CD patients, respectively. TRPV3 has been reported to be downregulated in UC mucosa and in the peripheral mononuclear cells of IBD patients [60,61]; however, relatively few studies exist in the literature regarding its role in IBD. From our enrichment analysis results, TRPV3 was correlated with genes involved in vitamin transport and metabolism, especially cobalamin (B-12), a finding which is consistent with the upregulated expression of this gene in the ileum of HIs (Figure 4) [62], which could warrant further research. Similarly, CNR1 has been documented to be downregulated in CD [63], and its activation confers protection against colitis by reducing inflammation [64,65]. Here, CNR1 together with S1PR4 formed a network with downregulated genes in the non-inflamed CD ileum, which participate in the proliferation and activation of B-cells. Even though CNR1 is known to be expressed in B-cells [66], its potential role in B-cell regulation in IBD has not yet been explored.

Finally, CHRM4 and the sphingosine-1-phosphate receptors (S1PRs) S1PR1, 3 and 4, exhibited dysregulated expression in either CD, UC, or both. CHRM4 was upregulated in the non-inflamed CD ileum and downregulated in UC, mainly in the ileum. According to the Human Protein Atlas, CHRM4 is expressed in the gastrointestinal tract and predominantly in the small intestine (https://www.proteinatlas.org/ENSG00000180720-CHRM4/tissue, accessed on 20 September 2023), which is also in line with our results regarding its upregulation in the ileum when compared the to colon (Figure 4). Although CHRM4 activation by acetylcholine in goblet cells has been demonstrated to regulate microbial sensing and antigen presentation in mice, and that the disruption of this process can lead to an inflammatory response [67], the role of this receptor in the pathophysiology of IBD is currently unknown.

In contrast, S1PRs have a well-studied role in the regulation of immune cell trafficking, and the S1PR1/5 modulator ozanimod is already in clinical use for the treatment of UC [68]. However, few studies exist regarding the expression of these receptors in IBD. Suh et al. have reported that S1PR1, S1PR2, and S1PR4 were upregulated in intestinal biopsies from IBD patients when compared to healthy controls [69], while Abarca-Zabalía et al. have shown a downregulation of the S1PR1 receptor in circulating CD4^+^ lymphocytes from CD patients [70]. Interestingly, the expression of S1PR1 was found to be induced by chronic and not acute inflammation in a DSS model of colitis in mice [71], which could offer some explanation for the inconsistencies in the findings regarding the expression of this receptor in IBD. In this study, we have shown that S1PR1 and S1PR4 are upregulated in the inflamed colonic mucosa in UC, S1PR3 is downregulated in the UC ileum, and S1PR4 is also upregulated in the inflamed colon and downregulated in the non-inflamed CD ileum. We also found that S1PR1, as well as S1PR3 and S1PR4, are upregulated in the presence of inflammation when compared to non-inflamed intestinal regions in IBD.

In addition to the known function of these receptors in immune cell trafficking [72], we demonstrate an association of S1PR1 and S1PR4 with the negative regulation of B-cell proliferation and the negative regulation of IL-10 production, respectively, in UC. Furthermore, a novel association of S1PR4 with the positive regulation of extracellular matrix disassembly in the CD colon was also found, rendering a potential target for future antifibrotic therapies in IBD.

Even though the therapeutic potential of MRs remains largely unexplored, there have been reports of IBD treatment via MR targeting, with the most successful being that of the S1PR modulator ozanimod, which is currently being used in clinical settings for the treatment of moderate to severe UC [68]. Furthermore, the anti-inflammatory effect of 5-aminosalicylic acid (5-ASA), a well-known, commonly used treatment for UC, has been recently attributed to the activation of PPAR-γ in the intestinal epithelium [42], implicating this receptor in the pathophysiology and treatment of this disease. The potential of the nuclear receptors FXR, PXR, and PPAR-γ as therapeutic targets for IBD has been recently reviewed by Ning et al. [73], and several chemical agents already in clinical use for other diseases have been found to regulate inflammation and tissue repair in IBD via MR activation, such as rosiglitazone and pioglitazone through PPAR-γ, and rifaximin and rifampicin though PXR.

In conclusion, the landscape of metabolite receptors is altered in patients with IBD, and the observed alterations are also dependent on the status of mucosal inflammation. Additionally, metabolite receptor expression is highly correlated with genes that exhibit dysregulated expression in IBD and those that participate in the pathophysiological processes of inflammation and fibrosis. Our study offers an insight into the perturbation of host microbial sensing in IBD, revealing possible mechanisms that could contribute to the development of disease and identify possible druggable targets for future therapies.

## 4. Materials and Methods

### 4.1. Data Acquisition and Processing

**Figure 6:**
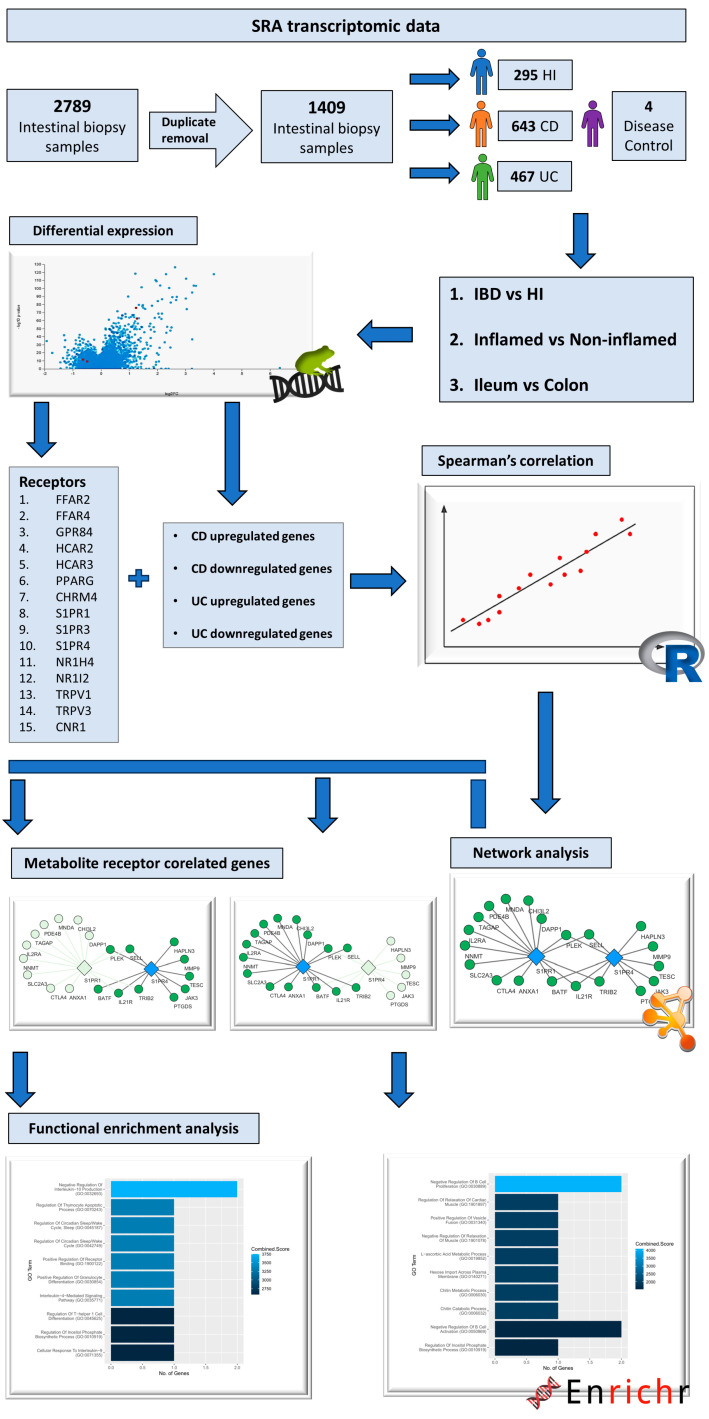
Flowchart depicting the steps followed and the tools used for this study.

Transcriptomic data derived from the RNA-sequencing of intestinal biopsy samples were collected from the public repository Gene Expression Omnibus (GEO) [74], using the following as query keywords: “(Inflammatory bowel disease OR Crohn’s disease OR Ulcerative colitis) AND (RNA sequencing OR transcriptome OR gene expression) AND biopsy”. The selection of datasets was based on the following criteria: 1. they contain RNA-sequencing raw data; 2. they include patients with CD and/or UC and/or healthy individuals (HIs); and 3. the data are derived from intestinal mucosa biopsy samples. In total, 5 datasets that matched our criteria were retrieved, 2 consisting of both patients with CD and UC, as well as healthy individuals (PRJNA797175 [75], PRJNA565216 [76]), 1 containing samples from CD patients and healthy individuals (PRJNA276116 [77]), 1 with samples from only patients with CD (PRJNA718848 [78]), and 1 with samples from only UC patients (PRJNA420681). The total amount of biopsy samples pooled from these datasets was 2785. After the removal of duplicate samples, meaning that every patient contributed 1 sample to the dataset, the number of samples remaining was 1405, corresponding to 295 healthy individuals, 643 Crohn’s disease patients, and 467 patients with ulcerative colitis. The RNA-sequencing dataset used to test the effects of non-specific inflammation on the expression of MRs (PRJNA348339 [79]) consisted of colonic biopsy samples which were derived form 4 *Campylobacter jejuni*-infected patients and 6 uninfected individuals. Information regarding the datasets used, and patient metadata can be found in Table S2 in Supplementary Materials. The study methodology is delineated in the flowchart of Figure 6.

### 4.2. Differential Expression Analysis

Gene differential expression was analyzed using the online platform RaNA-Seq, following the quantification and quality control assessment of samples [80]. Alignment on the GRCh38 reference genome and the quantification of reads was performed using Salmon [81], while plots produced via RJSplot [82] were utilized for sample quality control and curation. The differential expression analysis was performed with the DESeq2 algorithm [83], and differentially expressed metabolite receptor genes were selected based on the following criteria: adjusted *p* value < 0.05, and fold regulation lower than −1.5 and higher than 1.5. Several differential expression analyses were performed, and can be grouped into the three following categories: 1. inflammatory bowel disease vs. healthy individuals, 2. inflamed intestinal regions vs. non-inflamed, and 3. the ileum vs. the colon. Separate sub-analyses accounting for different intestinal regions (ileum/colon) and/or inflammation status (inflamed/non-inflamed) were accordingly performed for every category.

### 4.3. Correlation and Network Analysis

For the correlation analysis, the metabolite receptor genes and upregulated and/or downregulated genes that were identified as differentially expressed between IBD patients and HIs in each analysis were chosen, and their expression was correlated separately for IBD patients and HIs using Spearman’s correlation coefficient. The analysis was performed on R v4.3.0 with the standard “stats” package. Correlation matrices produced from these analyses were converted into network files, using gene names as nodes and correlation coefficients as edge weights. Network construction and analysis was performed using Cytoscape v3.10.0 [84].

### 4.4. Functional Enrichment Analysis

The up- or downregulated genes that highly correlated with each metabolite receptor in each analysis were used as input, and functional enrichment was performed using the Enrichr online platform. Visualizations of the results were produced using R v4.3.0 and the package “ggplot2”.

## Data Availability

Data is contained within the article and Supplementary Material.

## Supplementary Materials

The following supporting information can be downloaded at https://www.mdpi.com/article/10.3390/ph17040492/s1, Table S1: Differential expression of all the genes included in this study; Table S2: Datasets and patient metadata. Figure S1: Panel showing the top GO processes (based on combined score) associated with each MR in CD, derived from the functional enrichment of their strongly associated up- and/or down-regulated genes; Figure S2: Panel showing the top GO processes (based on combined score) associated with each MR in UC, derived from the functional enrichment of their strongly associated up- and/or down-regulated genes; The full resolution figures of this article can be found online at figshare following this DOI: 10.6084/m9.figshare.25464220.
