# Supplementary figures and images for: Exploring Microbial Metabolite Receptors in Inflammatory Bowel Disease: An In Silico Analysis of Their Potential Role in Inflammation and Fibrosis
Source: Pharmaceuticals (Basel). 2024 Apr 12;17(4):492. doi: 10.3390/ph17040492 (PMC11054721; doi:10.3390/ph17040492)

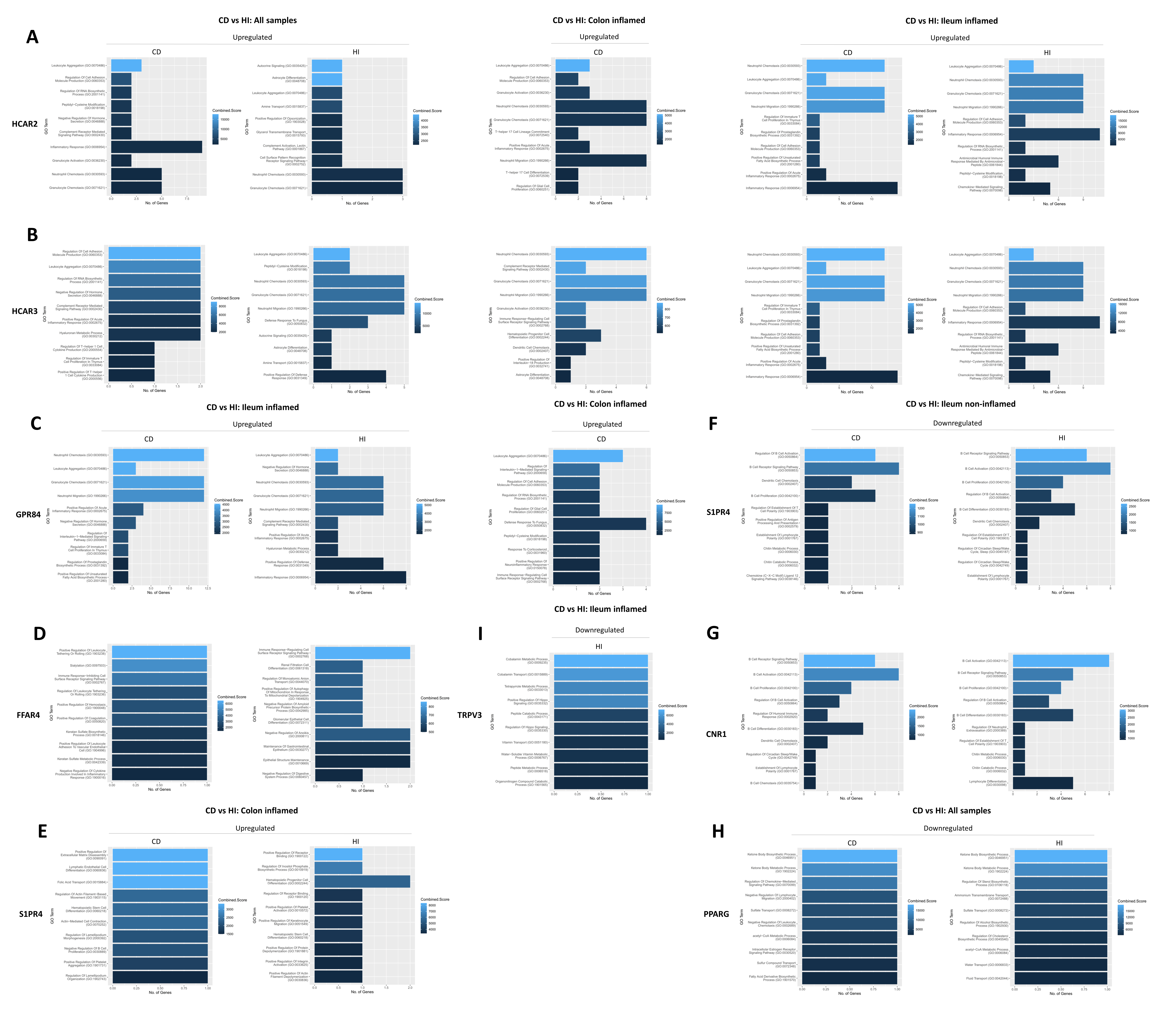

Supplement: Supplementary file 1 [file pharmaceuticals-17-00492-s001.zip › Supplementary Figure S1.tif]

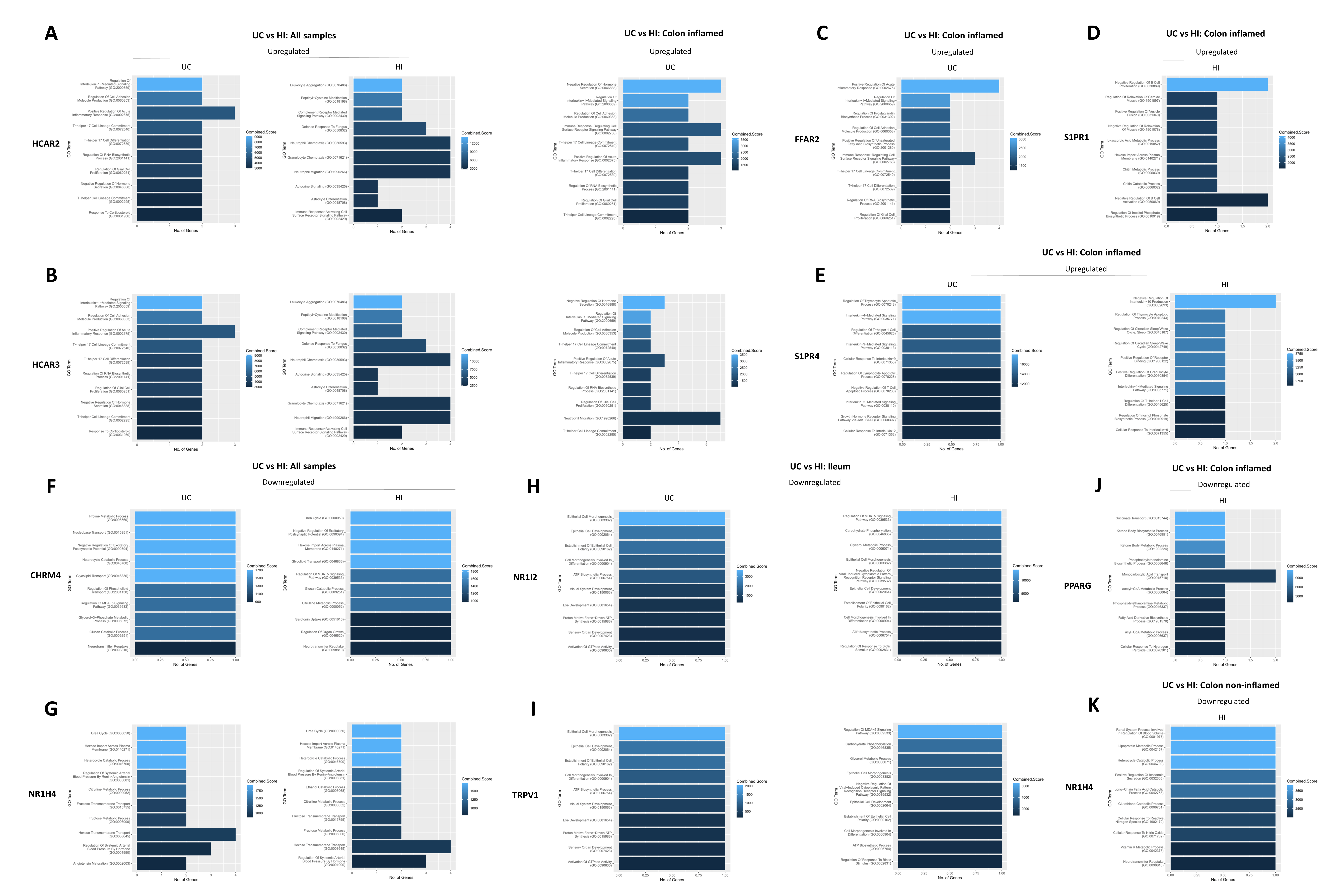

Supplement: Supplementary file 1 [file pharmaceuticals-17-00492-s001.zip › Supplementary Figure S2.tif]
